# The Remodeling Effects of High-Concentrate Diets on Microbial Composition and Function in the Hindgut of Dairy Cows

**DOI:** 10.3389/fnut.2021.809406

**Published:** 2022-02-01

**Authors:** Ruiyang Zhang, Junhua Liu, Linshu Jiang, Xinfeng Wang, Shengyong Mao

**Affiliations:** ^1^College of Animal Science and Veterinary Medicine, Shenyang Agricultural University, Shenyang, China; ^2^Laboratory of Gastrointestinal Microbiology, Jiangsu Key Laboratory of Gastrointestinal Nutrition and Animal Health, College of Animal Science and Technology, Nanjing Agricultural University, Nanjing, China; ^3^Beijing University of Agriculture, Beijing, China; ^4^College of Animal Science and Technology, Shihezi University, Shihezi, China

**Keywords:** high concentrate diets, hindgut microbiota, microbial function, dairy cows, metagenomics

## Abstract

At present, research on high-concentrate (HC) diets mostly focused on the rumen, and there is a paucity of information on the hindgut microbiota of dairy cows. In the present study, a 2 × 2 crossover design with four healthy Holstein cows was used, and the metagenomics approach was adopted to reveal the remodeling effects of HC diets on hindgut microbiota and their metabolic functions. Results showed that, compared with the low-concentrate (LC) diets, HC diets have markedly decreased (*p* < 0.05) the abundance of cellulolytic bacteria (such as *Fibrobacter, Ruminococcus*, and *Ruminiclostridium*) and methanogens (such as *Methanobrevibacter, Methanosarcina*, and *Methanosphaera*); and correspondingly, HC diets have significantly reduced (*p* < 0.05) the abundance of carbohydrate-active enzymes (CAZy) related to hemicellulases (GH10, GH11, and GH54) and cellulases (GH1, GH44, and GH45) and increased the abundance of one oligosaccharide-degrading enzyme (GH32). Furthermore, 62 Kyoto Encyclopedia of Genes and Genomes (KEGG) pathways of hindgut microbiota were affected (*p* < 0.05) by different dietary treatments, and the major pathways altered by HC diets were “Methane metabolism” (enriched in the LC group), “Lipid metabolism” (enriched in the HC group), and several sub-pathways in “Amino acid metabolism” (such as Phenylalanine metabolism, and Phenylalanine, tyrosine, and tryptophan biosynthesis). Also, the microbial genes involved in the pathways “Methane metabolism” (except 1 gene), “Tryptophan metabolism”, and “Phenylalanine metabolism” were all decreased (*p* < 0.05) in the present study. These findings suggested that HC diets caused the remodeling of hindgut microbiota and its potential functions, and these results may benefit in gaining a deeper understanding of the impact of HC diets on the hindgut microbiota of dairy cows.

## Introduction

For ruminants, the gastrointestinal tract-dwelling microbiota is central to nutrient digestion, production performance, and host health (1, 2). To maintain the high milk yields or growth rate, the current ruminant industry imposes a high-concentrate (HC) feeding pattern; this also induces gastrointestinal microdysbiosis, thus resulting in some health problems, such as subacute ruminal acidosis (SARA) (3, 4). Being the initial and primary organ of microbial fermentation, alterations in the ruminal microbiome of dairy cows after HC feeding have received much research attention (5, 6). Our previous study and others showed that the undegraded starch escaped from the rumen and the small intestine could also reach the hindgut under the HC feeding condition, which enabled to affect the internal environment and microbial structure in the hindgut of dairy cows (7, 8). However, compared with the ruminal microbiome, there is relatively limited information about the effects of HC feeding on the hindgut microbiome.

Previous studies demonstrated the events that occurred in the rumen mirrored in the hindgut after HC feeding, which also resulted in an acidic environment with potential pathogenic factors in the hindgut, specifically manifested as low pH, high levels of volatile fatty acids (VFAs), and lipopolysaccharides in feces of dairy cows (8–10). However, as compared with the multilayer squamous epithelium of rumen, the single-layer columnar epithelium of the hindgut is more fragile and susceptible to this potentially dangerous environment, which further damaged the host health and production performance (11, 12). Thus, the above findings indicate that HC feeding may alter the microbial composition and its metabolic functions in the hindgut of dairy cows. In addition to the host health, the enteric microbiota of dairy cows is related to food safety, fecal pollution and is also identified as the source of various microbial contamination, such as soil and water reservoirs (13, 14). Hence, a better understanding of how HC diets affect the hindgut microbiome is important in reducing fecal pollution and the related subsequent environmental problems. Moreover, taking feces through the rectum is less damaging to dairy cows and easier to harvest than the rumen contents. Therefore, enriching the information about fecal microbiome and the remodeling effects of HC diets could contribute to host health and environmental optimization and provide the possibility to use the characteristic microbial changes to reflect the effects of HC diets on the host response of dairy cows in the future.

Nowadays, the application of metagenomics has largely advanced our knowledge of the gastrointestinal microbiome. However, published studies that investigated the effects of HC diets on the hindgut microbial changes in dairy cows were limited to the methods of quantitative PCR or 16S rRNA sequencing (15) and could not unravel the alterations in microbial structure and its potential metabolic functions synchronously. This study, therefore, aimed to reveal the effects of HC diets on the fecal microbiome and their overall metabolic functions using a metagenomics approach, and the results of the present study may benefit in gaining a deeper understanding of the impact of HC feeding on the hindgut microbiota of dairy cows.

## Materials and Methods

### Animals, Feeding Diets, and Experimental Design

The present study was part of a series of studies on the impact of HC diets on the gastrointestinal tract of dairy cows, and detailed and complete animal experimental protocols have been provided previously (16, 17). In brief, the 2 × 2 crossover experimental design (two treatments and two periods) was applied in the present study. Four healthy and lactating Holstein dairy cows (460 ± 16.4 kg body weight and 84 ± 25 days in milk) with long-established fistulas were randomly allocated to the following two different experimental diets: low-concentrate (LC) diets (40% concentrate feed, DM) or HC diets (70% concentrate feed, DM). The detailed ingredients and nutrition levels of experimental diets in the present study are presented in Supplementary Table S1. Before the experiment began, all cows were fed LC diets (30% concentrate feed, DM) for 3 weeks. For cows fed HC diets, the dietary concentrate level was gradually increased to 70% in the first 2 days. During the whole feeding period, a total mixed ration was available *ad libitum* for experimental dairy cows and free access to water. In each experimental period, the dairy cows were fed the corresponding treatment diets for 21 days, and fecal samples were harvested by the rectal method at 4 h after morning feeding on the last day of each period.

The collected samples were stored in an −80°C experimental refrigerator until DNA extraction was performed.

### DNA Extraction, Pyrosequencing, and Functional Annotations

Metagenomic DNA was isolated utilizing a QIAamp DNA Mini Kit (Qiagen, Valencia, CA, USA) in accordance with the standard extraction protocol provided by the manufacturer. To obtain efficiently extracted quality DNA from fecal samples, the bead-beating process was adopted to mechanically break the microbial cell wall. The above-extracted DNA has measured the extracted quality and quantified concentration by a Nanodrop Spectrophotometer (Nyxor Biotech, Paris, France). Finally, DNA samples were stored at −80°C until subsequent assays.

Total genomic DNA was fragmented by the Covaris M220 Focused-ultrasonicator (Covaris Inc., Woburn, MA, USA), and the sequencing libraries were constructed using a TruSeq™ DNA Sample Prep Kit (Illumina, San Diego, CA, USA) and sequenced on an Illumina HiSeq PE 150 platform. To harvest the clean data, the quality control of original sequencing data was conducted to remove adapters and low-quality reads using Trimmomatic (18). The retained reads were then mapped to *Bos taurus* ARS-UCD1.2 to remove the host-genome contaminations through the Burrows–Wheeler Aligner (BWA, V0.7.12) (19). The metagenome assembles of clean data were conducted by Megahit (V1.1.1) (20). Only the assembled contigs longer than 500 bp were adopted for subsequent analysis. The open reading frame (ORF) prediction was performed using the software MetaGeneMark (V2.10) (21), and the CD-HIT (V4.5.8) (22) was adopted to remove redundancy and constructed the initial gene catalog. The species annotations were harvested by blasting the Unigenes with the Non-Redundant (NR) database in the National Center for Biotechnology Information (NCBI). For further analysis, the Unigenes were blasted with the Kyoto Encyclopedia of Genes and Genomes (KEGG) and the Carbohydrate-active Enzymes (CAZy) to harvest the functional annotations information.

### Statistical Analysis

Statistics on microbial taxonomic data and the relative abundance of KEGG Orthology (KO) genes and CAZy were conducted using the general linear model (GLM) in IBM SPSS statistics V20.0.0 (IBM Corp., Armonk, NY, USA). The statistical model applied in the present study was as follows: *y*_*i*_ = μ + *S* + *P* + SP + *e*_*i*_. Where μ represents the overall mean, *S* represents the dietary treatment, *P* represents the fixed effect of the experimental period, SP represents the interactions between the diet treatment and period, and *e*_*i*_ represents the random residual. The correction of values of *p* obtained by the above statistical model was performed through the false discovery rate (FDR), and the corrected *p* < 0.05 was defined as significant.

The principal coordinate analysis (PCoA) and the analysis of molecular variance (AMOVA) were run within R software packages (23) to evaluate the sample distribution and statistical differences between the groups. To identify the characteristic changes at the species level in fecal samples from different dietary treatments, the linear discriminant analysis effect size (LEfSe) was conducted. The hierarchical analysis of KEGG pathways (level 3) was conducted with the Number Cruncher Statistical System (NCSS, version 12.0.2; Kaysville, UT, USA).

## Results

### Microbial Ecology of the Fecal Microbiota in Dairy Cows

**Figure 1:**
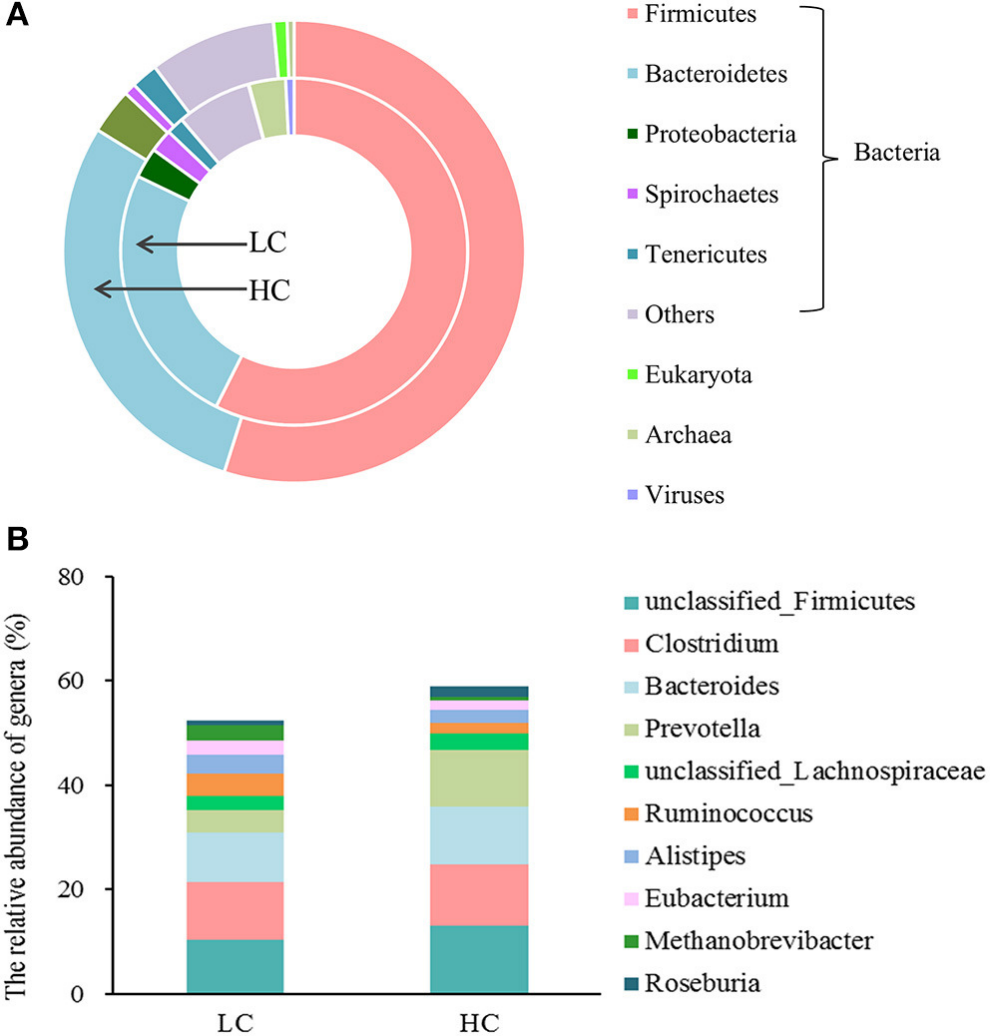
The overview of the fecal microbial composition of dairy cows at the level of the kingdom **(A)** and genus **(B)**. Only the 5 most abundant phylum and the 10 most abundant genera in bacteria were shown in the figure.

At the domain level (Figure 1A), the fecal microbial community in the LC and HC groups was dominated by bacteria (95.73 vs. 98.55%), followed by archaea (3.41 vs. 0.94%) and viruses (0.79 vs. 0.49%), and eukaryote was the least with <0.1% relative abundance (0.07 vs. 0.02%). At the phylum level, the fecal microbial community in the LC and HC groups was dominated by Firmicutes (57.43 vs. 54.89%), Bacteroidetes (24.72 vs. 28.93%), Proteobacteria (2.83 vs. 3.17%), Spirochaetes (2.15 vs. 0.83%), and Tenericutes (1.63 vs. 1.87%). At the genus level (Figure 1B), unclassified *Firmicutes* (10.25 vs. 13.08%), *Clostridium* (11.18 vs. 11.62%), *Bacteroides* (9.46 vs. 11.12%), *Prevotella* (4.24 vs. 10.79%), unclassified *Lachnospiraceae* (2.85 vs. 3.36%), *Ruminococcus* (4.12 vs. 2.04%), *Alistipes* (3.73 vs. 2.30%), *Eubacterium* (2.62 vs. 1.93%), *Methanobrevibacter* (3.01 vs. 0.61%), and *Roseburia* (0.84 vs. 2.17%) were dominated in the fecal microbial community in the LC and HC groups.

### The Fecal Microbial Community in HC-Fed Cows

**Figure 2:**
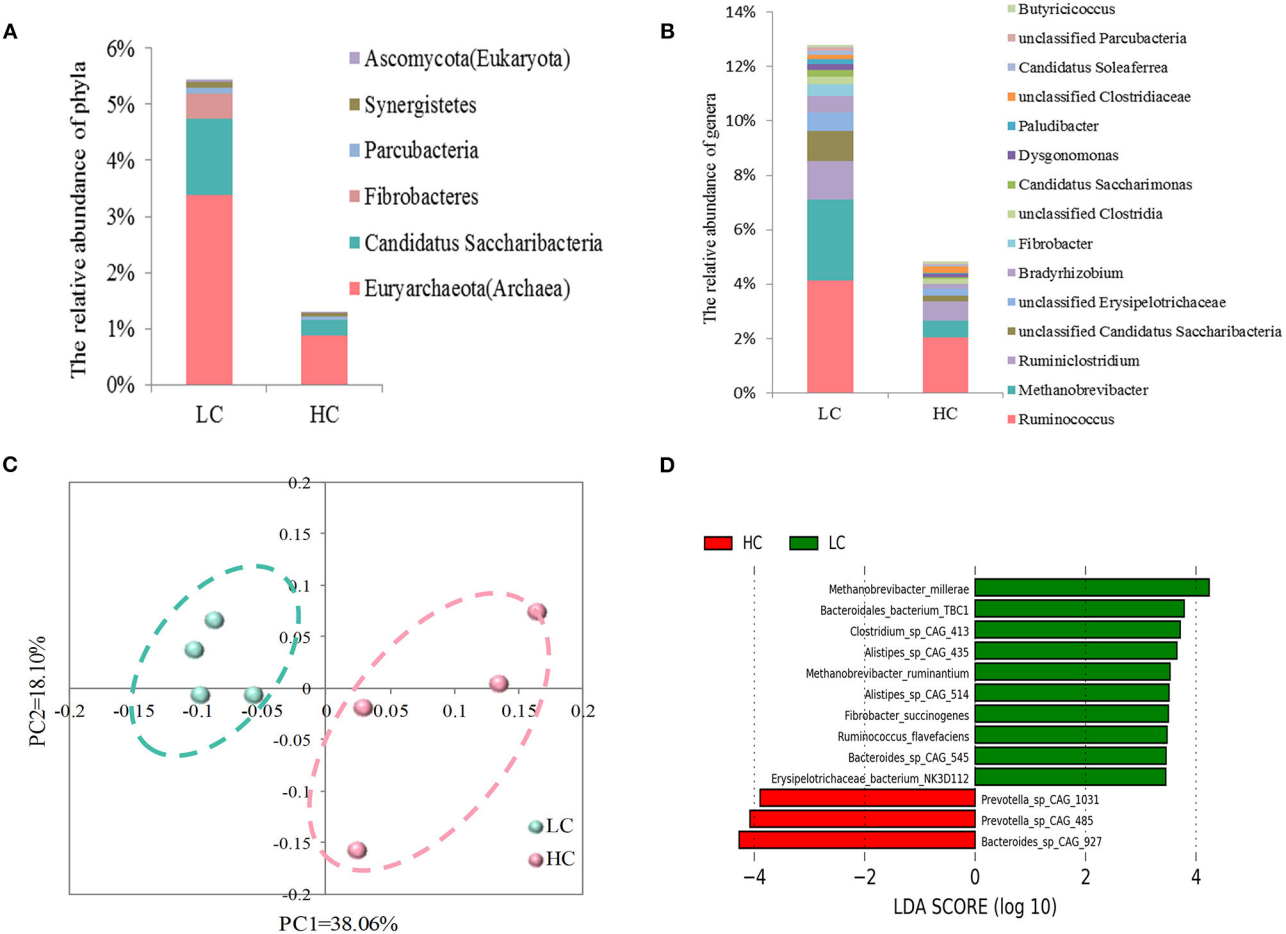
Effects of high-concentrate (HC) diets on the fecal microbial communities in dairy cows at different taxonomic levels. Comparison of the fecal microbial communities at the level of phylum **(A)** and genus **(B)**. Only the phyla and genera that were significantly affected by dietary treatments were presented. **(C)** The principal coordinate analysis (PCoA) of fecal microbial functions at the species level. **(D)** Histogram of linear discriminant analysis (LDA) scores for abundant species between the LC and HC groups.

Through the metagenomics analysis, we evaluated the effects of HC diets on the fecal microbial communities at different taxonomic levels. Of phyla (>0.05%; Figure 2A), a decrease in the relative abundance of *Euryarchaeota* (Archaea; *p* = 0.012), *Candidatus Saccharibacteria* (*p* = 0.014), *Fibrobacteres* (*p* = 0.021), *Parcubacteria* (*p* = 0.024), *Synergistetes* (*p* = 0.027), and *Ascomycota* (Eukaryota; *p* = 0.024) were found in HC-fed cows compared with LC-fed cows. Of genera (>0.1%) showing significant (Figure 2B), a decrease in the relative abundance of *Ruminococcus* (*p* = 0.033), *Methanobrevibacter* (Archaea; *p* = 0.028), *Ruminiclostridium* (*p* = 0.031), *Bradyrhizobium* (*p* = 0.033), *Fibrobacter* (*p* = 0.033), *Candidatus Saccharimonas* (*p* = 0.019), *Dysgonomonas* (*p* = 0.041), *Paludibacter* (*p* = 0.034), *Candidatus Soleaferrea* (*p* = 0.036), *Butyricicoccus* (*p* = 0.032), and 4 unclassified genera and an increase in unclassified *Clostridiaceae* (*p* = 0.049) were observed in HC-fed cows when compared with LC-fed cows. Besides, we also found the relative abundance of genera *Methanosarcina* and *Methanosphaera* (<0.1%, data not shown), belonging to *Archaea*, was also decreased (*p* < 0.05) by HC diets compared with LC diets.

At the species level, the results of PCoA (Figure 2C) revealed that the LC samples were separated clearly from HC samples without any overlap, and the statistical analysis of AMOVA showed that this difference in LC and HC groups was significant (Fs = 3.164, *p* = 0.032). Then, LEfSe was adopted to identify the key species responsible for the differences between the LC and HC groups (Figure 2D). *Bacteroides sp. CAG: 927, Prevotella sp. CAG: 485*, and *Prevotella sp. CAG: 1031*, which were enriched in the HC group, and *Methanobrevibacter millerae* (Archaea), *Methanobrevibacter ruminantium* (Archaea), *Bacteroidales bacterium TBC1, Bacteroides sp. CAG: 545, Clostridium sp. CAG: 413, Alistipes sp. CAG: 514, Alistipes sp. CAG: 435, Fibrobacter succinogenes, Ruminococcus flavefaciens*, and *Erysipelotrichaceae bacterium NK3D112*, which were enriched in the LC group, were the dominant species that contributed to the microbial differences between the two groups.

### Alterations in the Profiles of CAZy After HC Feeding

**Figure 3:**
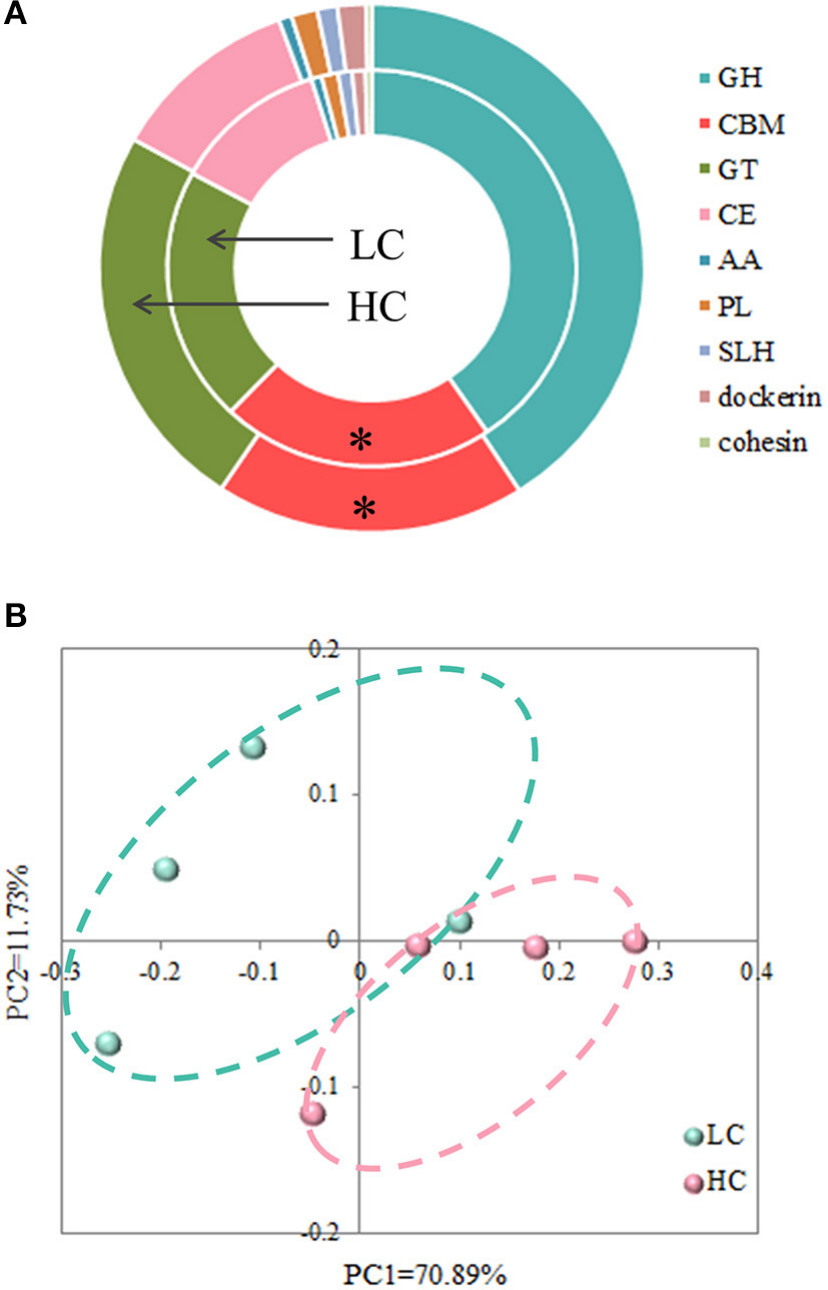
Effects of high-concentrate (HC) diets on profiles of carbohydrate-active enzymes at the class **(A)** and phylum **(B)** levels. *p < 0.05.

Due to the degradation process of carbohydrates requiring the participation of multiple enzymes, we focused on analyzing the differences in the profiles of CAZy between the LC- and HC-fed cows. At the class level (Figure 3A), the carbohydrate-binding module (CBM) was less abundant (*p* = 0.023) in the fecal microbiota of HC-fed cows compared with those in LC-fed cows. However, the dietary treatments did not affect the relative abundance of glycoside hydrolases (GH), glycosyltransferases (GT), carbohydrate esterases (CE), auxiliary activity (AA), polysaccharide lyases (PL), S-layer homology domain (SLH), dockerin, and cohesion.

At the phylum level, the PCoA with Bray-Curtis distance (Figure 3B) suggested that the LC group was approximately separated from the HC group, and AMOVA analysis confirmed that these differences in the two groups were reached at statistical tendency (Fs = 3.26747, *p* = 0.085). For further understanding of the effects of dietary treatments on CAZy, we conducted a statistical analysis of the relative abundance of enzymes (Table 1). The results revealed that there were 24 abundant enzymes that (>0.01% relative abundance) were affected by HC diets, among them, the relative abundance of 1 oligosaccharide-degrading enzyme (GH32), 2 N-acetylglucosaminidase (GH85 and GH101), 1 mycodextranase (GH87), and 3 GTs (GT20, GT39, and GT83) were increased (*p* < 0.05) by HC diets when compared with LC diets. Meanwhile, the remaining 21 abundant enzymes, i.e., 1 oligosaccharide-degrading enzyme (GH17), 3 endo-hemicellulases (GH10, GH11, and GH54), 2 cellulases (GH1, GH44, and GH45), 1 N-acetylmuramidase (GH108), 3 CBMs (CBM16, CBM44, and CBM59), 3 CEs (CE12, CE13, and CE15), 2 PLs (PL9 and PL14), and 1 AA (AA4) were decreased (*p* < 0.05) by HC diets when compared with LC diets.

**Table 1:** The effects of high-concentrate (HC) diets on the relative abundance of abundant carbohydrate-active enzymes (>0.01% in at least one group) in the fecal samples of dairy cows.

**Enzyme classes**	**Enzyme family**	**Major activity**	**LC (%)**	**HC (%)**	**SEM**	***P-*value**
**Glycoside hydrolases**	**Oligosaccharide-degrading enzymes**					
	GH17	Glucan endo-1,3-β-glucosidase,glucan 1,3-β-glucosidase	0.01	0.00	0.002	0.022
	GH32	Invertase, endo-inulinase	0.26	0.37	0.030	0.032
	**Endo-hemicellulases**					
	GH10	Endo-1,4-β-xylanase,endo-1,3-β-xylanase	0.42	0.23	0.050	0.029
	GH11	Endo-β-1,4-xylanase,endo-β-1,3-xylanase	0.02	0.00	0.005	0.023
	GH54	α-L-arabinofuranosidase, β-xylosidase	0.02	0.00	0.006	0.042
	**Cellulases**					
	GH1	β-glucosidase,β-galactosidase	0.34	0.16	0.053	0.019
	GH44	Endoglucanase, xyloglucanase	0.03	0.00	0.006	0.017
	GH45	Endoglucanase, endo-xyloglucanase	0.01	0.00	0.003	0.046
	**Others**					
	GH85	Endo-β-N-acetylglucosaminidase	0.02	0.17	0.035	0.049
	GH101	Endo-α-N-acetylgalactosaminidase	0.00	0.01	0.003	0.021
	GH108	N-acetylmuramidase	0.06	0.02	0.012	0.030
	GH87	Mycodextranase,α-1,3-glucanase	0.00	0.01	0.002	0.025
**Carbohydrate-binding modules**						
	CBM16	Binding to cellulose and glucomannan	0.93	0.35	0.132	0.047
	CBM44	Binding to cellulose and xyloglucan	0.35	0.19	0.043	0.048
	CBM59	Binding to mannan, xylan, and cellulose	0.01	0.00	0.003	0.032
**Carbohydrate esterases**	CE12	Pectin acetylesterase, rhamnogalacturonan acetylesterase	0.46	0.31	0.035	0.030
	CE13	Pectin acetylesterase	0.02	0.01	0.005	0.008
	CE15	4-O-methyl-glucuronoyl methylesterase	0.23	0.09	0.029	0.028
**GlycosylTransferases**	GT20	α,α-trehalose-phosphate synthase [UDP-forming], Glucosylglycerol-phosphate synthase	0.00	0.01	0.002	0.021
	GT39	Dol-P-Man: protein α-mannosyltransferase	0.10	0.29	0.049	0.018
	GT83	Undecaprenyl phosphate-α-L-Ara4N: 4-amino-4-deoxy-β-L-arabinosyltransferase	0.13	0.28	0.030	0.017
**Polysaccharide lyases**	PL14	Alginate lyase, exo-oligoalginate lyase	0.01	0.00	0.003	0.019
	PL9	Pectate lyase, exopolygalacturonate lyase	0.20	0.08	0.026	0.021
**Auxiliary activities**	AA4	Vanillyl-alcohol oxidase	0.09	0.04	0.010	0.018

*Only the significantly affected enzymes were presented in the table*.

### Differences in KEGG Profiles of Fecal Microbiota After HC Feeding

**Figure 4:**
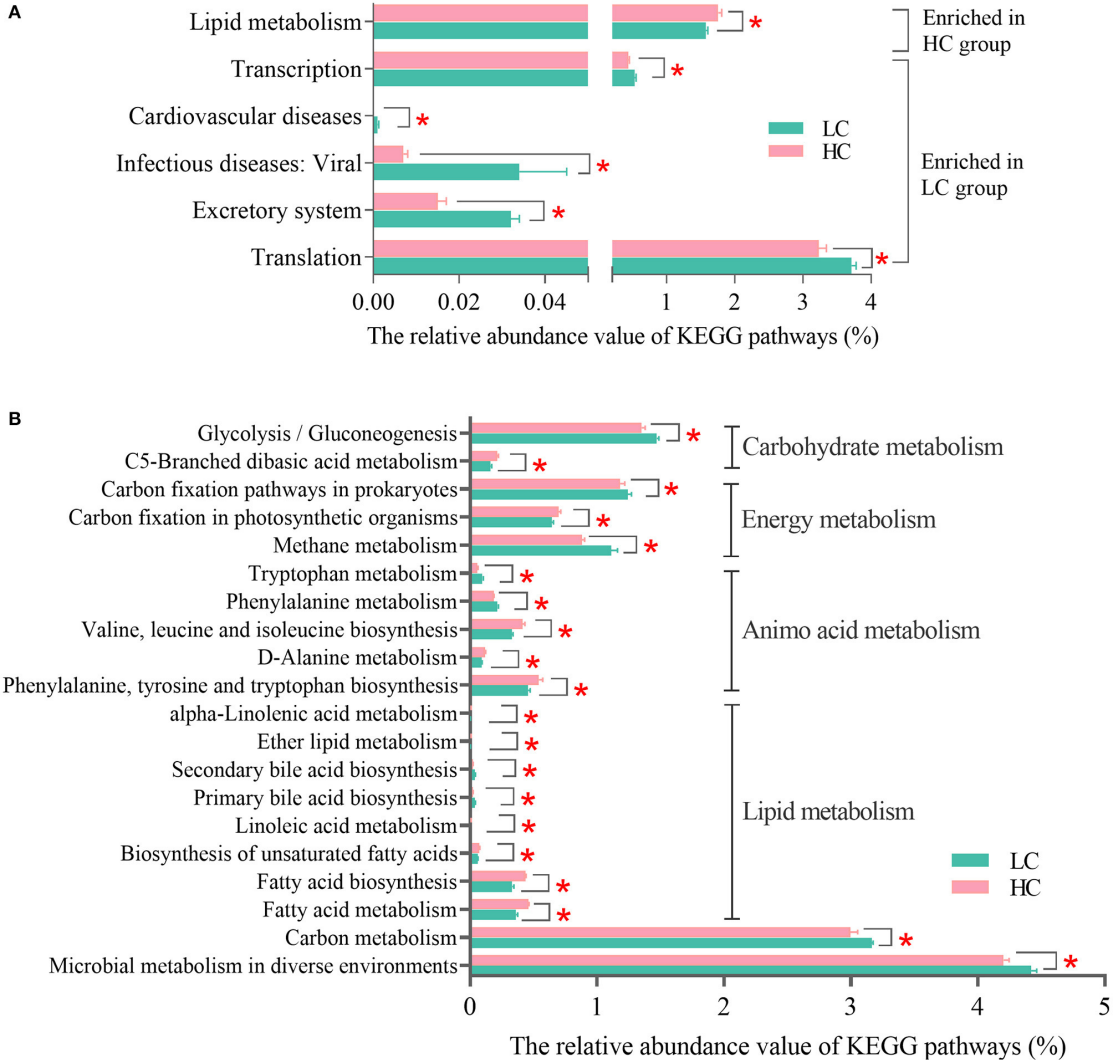
Effects of high-concentrate (HC) diets on KEGG profiles of the fecal microbiota of dairy cows at level 2 **(A)** and level 3 **(B)**. KEGG, Kyoto Encyclopedia of Genes and Genomes. *p < 0.05.

For further understanding of how HC diets affected the microbial functions in the hindgut of dairy cows, the KEGG profiles (level 2 and level 3) were analyzed. At KEGG level 2 (Figure 4A), the results showed that the “Lipid metabolism” was enriched in the HC group, while the “Excretory system,” “Excretory system,” “Cardiovascular diseases,” “Transcription,” and “Translation” were enriched in the LC group. At the KEGG level 3, the results of PCoA and AMOVA (Supplementary Figure S1A) revealed that there were certain differences in the profiles of KEGG pathways between the samples of two groups in the present study (Fs = 7.00599, *p* = 0.067). There were 62 KEGG pathways affected by different dietary treatments (Supplementary Figure S1B), and after classification and re-carding, the main altered KEGG pathways are listed in Figure 4B. The results showed that two “Carbohydrate metabolism” pathways, three “Energy metabolism” pathways, five “Amino acid metabolism” pathways, eight “Lipid metabolism,” the pathway “Carbon metabolism,” and the pathway “Microbial metabolism in diverse environments” were affected by different dietary treatments in the present study. Among these main affected KEGG pathways, the pathways “Glycolysis/Gluconeogenesis,” “Carbon fixation pathways in prokaryotes,” “Methane metabolism,” “Tryptophan metabolism,” “Phenylalanine metabolism,” “Secondary bile acid biosynthesis”, and “Primary bile acid biosynthesis” were enriched in the LC group, and the other pathways were enriched in the HC group.

### Microbial Gene Abundance Analysis of Fecal Microbiota in HC-Fed Cows

**Figure 5:**
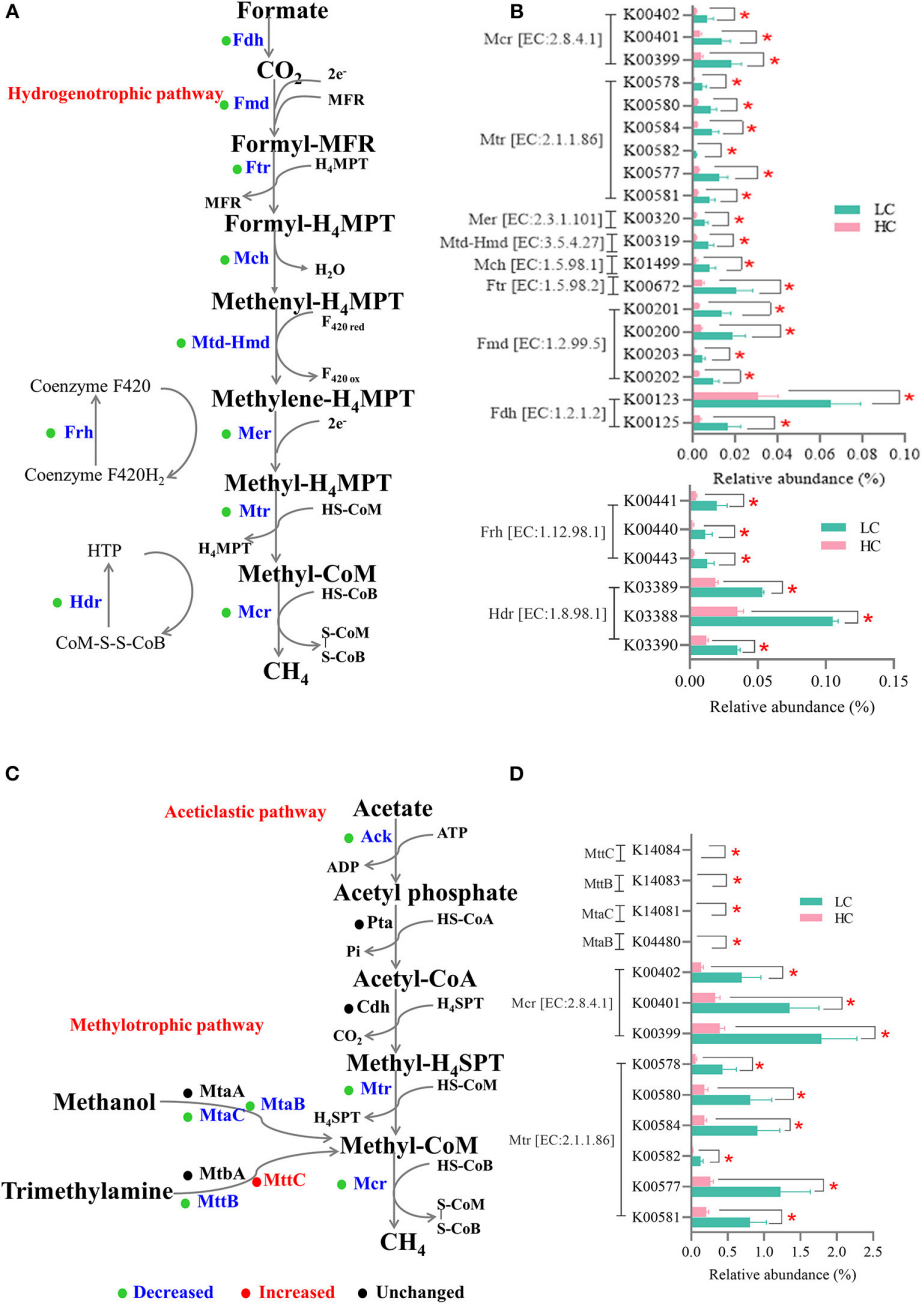
Effects of high-concentrate (HC) feeding on microbial genes involved in methanogenesis of dairy cows. The enzymes involved in the methanogenesis pathway—the hydrogenotrophic route **(A)** and the acetoclastic route, and the methylotrophic route **(C)**. The relative abundance of microbial genes involved in the hydrogenotrophic route **(B)** and the acetoclastic and methylotrophic route **(D)**. The blue font indicated that the relative abundance of this enzyme was significantly reduced in the HC group. *p < 0.05.

In the present study, the KO genes significantly affected by diets were imported into the KEGG Mapper for visualization, and the results showed that 67 differential genes were related to methane metabolism. Since the methane in the gastrointestinal tract is mainly produced through the hydrogenotrophic route (predominant), the acetoclastic route, and the methylotrophic route (24), and hence, we separately screened the genes involved in these three routes. As for the hydrogenotrophic route (Figures 5A,B), the relative abundance of all genes encoding catalytic enzymes related to the reduction reactions of CO_2_/H_2_ to methane, such as formate dehydrogenase (*Fdh*, EC: 1.2.1.2), formylmethanofuran dehydrogenase (*Fmd*, EC: 1.2.99.5), formylmethanofuran-tetrahydromethanopterin N-formyltransferase (Ftr, EC: 2.3.1.101), methenyltetrahydromethanopterin cyclohydrolase (*Mch*, EC: 3.5.4.27), methylenetetrahydromethanopterin dehydrogenase (*Mtd-Hmd*, EC: 1.5.98.1), 5,10-methylenetetrahydromethanopterin reductase (*Mer*, EC: 1.5.98.2), tetrahydromethanopterin S-methyltransferase (*Mtr*, EC: 2.1.1.86), and methyl-coenzyme M reductase (*Mcr*, EC: 2.8.4.1), was decreased (*p* < 0.05) by HC diets when compared with LC diets. In addition, the relative abundance of genes encoding coenzyme F420 hydrogenase (*Frh*, EC: 1.12.98.1) and heterodisulfide reductase (*Hdr*, EC: 1.8.98.1) was also decreased (*p* < 0.05) after HC-diets feeding when compared with LC-diets feeding. As for the acetoclastic route and the methylotrophic route (Figures 5C,D), in addition to genes encoding Mtr and Mcr, which were also presented in the hydrogenotrophic route, only the relative abundance of genes encoding methanol-5-hydroxybenzimidazolylcobamide Co-methyltransferase (*MtaB*), methanol corrinoid protein (*MtaC*), and trimethylamine-corrinoid protein Co-methyltransferase (*MttB*) was decreased (*p* < 0.05), and the gene encoding trimethylamine corrinoid protein (*MttC*) was increased (*p* < 0.05) after HC-diets feeding when compared with LC-diets feeding.

**Figure 6:**
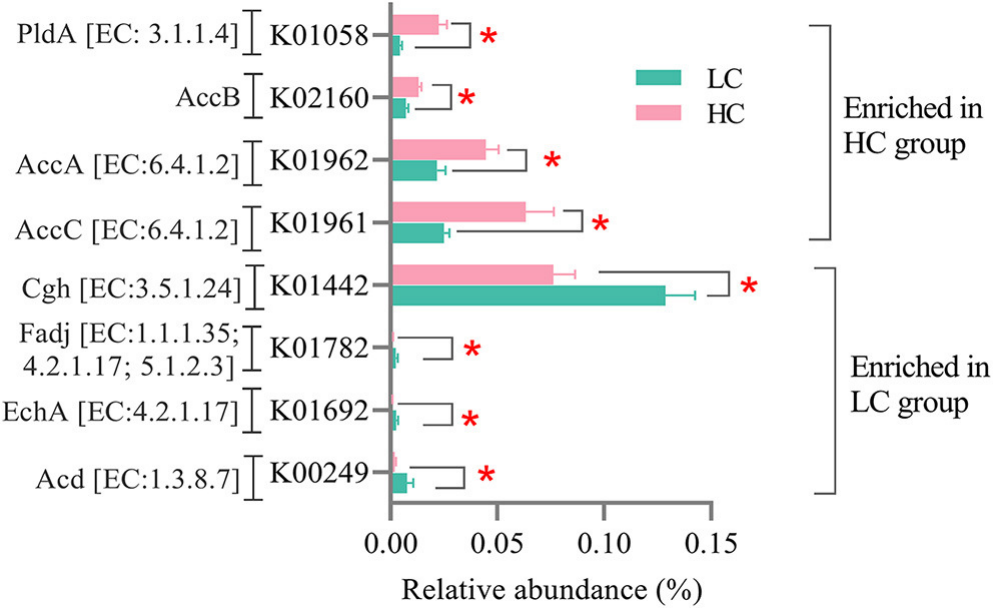
Effects of high-concentrate (HC) feeding on microbial genes involved in the lipid metabolism of dairy cows. *p < 0.05.

As for the microbial genes related to lipid metabolism (Figure 6), the relative abundance of genes encoding acyl-CoA dehydrogenase (*Acd*, EC: 1.3.8.7), enoyl-CoA hydratase (*EchA*, EC: 4.2.1.17), 3-hydroxyacyl-CoA dehydrogenase/enoyl-CoA hydratase/3-hydroxybutyryl-CoA epimerase (*Fadj*, EC: 1.1.1.35/4.2.1.17/5.1.2.3), and choloylglycine hydrolase (*Cgh*, EC: 3.5.1.24) was decreased (*p* < 0.05), whereas the relative abundance of genes encoding acetyl-CoA carboxylase, biotin carboxylase subunit (*AccC*, EC: 6.4.1.2), acetyl-CoA carboxylase carboxyl transferase subunit alpha (*AccA*, EC: 6.4.1.2), acetyl-CoA carboxylase biotin carboxyl carrier protein (*AccB*), and phospholipase A1 (*PldA*, EC: 3.1.1.32/3.1.1.4) was increased (*p* < 0.05) in the HC group when compared with the LC group.

For the microbial genes related to amino acid metabolism (Supplementary Figure S2) and glycolysis/gluconeogenesis (Supplementary Figure S3), the relative abundance of affected microbial genes related to the pathways “Tryptophan metabolism” and “Phenylalanine metabolism” was all decreased (*p* < 0.05) in the HC group when compared with the LC group. The relative abundance of fructose-bisphosphate aldolase (*Fba*, EC: 4.1.2.13), cyclohexadienyl/prephenate dehydrogenase (*TyrC*, EC: 1.3.1.43), chorismate mutase (*AroH*, EC: 5.4.99.5), chorismate mutase (*PheA*, EC: 5.4.99.5), and 3-dehydroquinate synthase II (*Dds*, EC: 1.4.1.24) in the pathway “Phenylalanine, tyrosine, and tryptophan biosynthesis” and the leucine dehydrogenase (*LeuDH*, EC: 1.4.1.9) and branched-chain amino acid aminotransferase (*IlvE*, EC: 2.6.1.42) in the pathway “Valine, leucine and isoleucine biosynthesis” was decreased (*p* < 0.05), while the relative abundance of the other affected microbial genes in these two pathways was increased (*p* < 0.05) in the HC group when compared with the LC group.

## Discussion

In the modern dairy farming industry, HC feeding was often used to maintain the high production performance of dairy cows, but some health problems ensued, and these problems were closely related to microbial fermentation and metabolism (3). Our previous study had confirmed that HC diets resulted in an increase in starch content in the hindgut of dairy cows, which in turn caused the accumulation of VFAs and pH decline (7). However, compared with the rumen, there is a paucity of information on the microbial composition and its metabolic functions in the hindgut of dairy cows after HC feeding. Hence, in the present study, a metagenomics approach was applied to ascertain the ability of HC diets to reshape the architecture (at the microbial phylum, genus, and species level) and functions (CAZy, KEGG pathways, and microbial genes) of hindgut microbiota in dairy cows. Our findings may provide a global understanding of the HC diets on microbial composition and functions in the hindgut of dairy cows.

### The Hindgut Adapts to HC Diets by Remodeling the Microbial Composition, Especially Cellulolytic Bacteria and Methanogens

After long-term co-evolution, the animal gastrointestinal tract has formed a stable and complex micro-ecosystem in which bacteria, archaea, eukaryotes, and viruses coexist (25). Our results showed that similar to the rumen microbial structure, bacteria occupied an absolutely dominant position (>95%), and archaea were in a subordinate position of the total microbes (25). At lower taxonomic levels, Firmicutes, Bacteroidetes, Proteobacteria, Spirochaetes, and Tenericutes were the five most abundant phyla, and unclassified Firmicutes, Clostridium, Bacteroides, Prevotella, and unclassified Lachnospiraceae were the five most abundant genera in the fecal microbial community of dairy cows.

Various factors have the effect of remodeling the microbial structure, with the diet and particularly dietary carbohydrates are key determinants for the composition and activity of the gastrointestinal microbiome (26). As expected, our results of Bray-Curtis PCoA and AMOVA indicated that there were significant differences in the fecal microbial community between the LC and HC groups. Indeed, we found some differences in the specific microorganisms between two groups at the levels of phylum, genus, and species, respectively. At the phylum level, a decrease in the abundance of Fibrobacteres, Parcubacteria, Synergistetes, Candidatus Saccharibacteria, Euryarchaeota (Archaea), and Ascomycota (Eukaryota) was found in the HC group when compared with that in the LC group. The phylum Fibrobacteres, its only genus *Fibrobacter*, and its cultured species *Fibrobacter succinogenes* are known as major cellulolytic degraders in the herbivore gut (27). Unsurprising, consistent with a previous study (28), the relative abundance of Fibrobacteres, *Fibrobacter*, and *Fibrobacter succinogenes* (as mentioned below) was all decreased by HC feeding. This decrease may be due to the increased starch content, decreased fiber content, and increased acidity caused by the changes in fermentable substrates entered into the hindgut (12, 28). Ascomycota, the abundant fungus in the rumen, was reported to proliferate in the rumen of dairy cows after HC feeding (29), while its relative abundance was decreased in the feces of dairy cows after HC feeding in the present study. Due to the limited information, the reason for this inconsistent result is different regions of the gastrointestinal tract or others still need further study. Euryarchaeota was the dominant phylum in the fecal archaeal community and comprised methanogenic bacteria species (30); the decreased relative abundance of this phylum in the present study indicated that HC feeding may affect methane production, but further analysis at lower taxonomic levels is required.

At lower taxonomic levels, the genera *Fibrobacter, Ruminococcus*, and their important species *F. succinogenes* and *R*. *flavefaciens*, which are considered as representative cellulolytic bacteria in the herbivore gut (31), were decreased by the HC diets in the present study. As mentioned above, these cellulolytic bacteria are more sensitive to the low pH caused by increased starch, and hence, their proliferation in the hindgut during HC feeding is inhibited. *Ruminiclostridium* species was demonstrated to be able to secrete extracellular multi-enzymatic complexes, which further efficiently decomposed the cellulose (32). In the present study, a decrease in the relative abundance of genus *Ruminiclostridium* was observed in the HC group, and this decrease may be also caused by the substrate changes in the hindgut of dairy cows. Interestingly enough, we also found the genera *Methanobrevibacter, Methanosarcina*, and *Methanosphaera* (belonging to archaea), and the important species (M. *millerae* and M. *ruminantium*) in *Methanobrevibacter*, were also decreased by the HC feeding in the present study. *Methanobrevibacter* is the most dominant archaeal genus in the rumen and feces of dairy cows (33, 34). These archaea members are responsible for methane production in the gastrointestinal tract of livestock animals (35), and hence, the decreased methanogen populations in the present study may result in less methane production from the gastrointestinal tract of dairy cows. Although we did not measure methane production in the present study, several previous studies had confirmed that increasing the proportion of concentrate in the diets could reduce methane production (36–38). Besides, there is a symbiotic relationship between methanogens and bacteria in the animal gut, most cellulolytic bacteria produced hydrogen during their metabolic process, and a positive correlation was observed between the number of cellulolytic bacteria and methanogens in the rumen of various ruminant species (39). Hence, the reduction in the relative abundance of cellulolytic bacteria in the present study may limit the available quantity of hydrogens that were converted to methane.

### The Relative Abundance of CAZy Associated With the Degradation of Cellulose and Hemicellulose Was Reduced by HC Feeding

The complex degradation process of carbohydrates in HC diets requires the participation of multiple enzymes, such as GH, PL, CE, GT, AA, and CBMs. The PCoA and AMOVA analysis of CAZy at the phylum level showed that HC diets had a certain effect on fecal microbial CAZy in dairy cows. Since the different content of cellulose and starch in dietary treatments in the present study, the degradation of cellulose, hemicellulose, and sugars attracted our special attention. GHs are a widespread and extremely important group of enzymes capable of hydrolyzing the glycosidic bonds that existed in carbohydrates, and CBMs possess the carbohydrate-binding activity and, hence, contribute to the degradation of carbohydrates (40). In the present study, the relative abundance of all GHs, which are classified as hemicellulases (GH10, GH11, and GH54) and cellulases (GH1, GH44, and GH45), were reduced after HC-diets feeding. Meanwhile, the relative abundance of all affected CBMs (CBM 16, CBM 44, and CBM59), which possessed cellulose-binding activity, was also reduced after HC-diets feeding. This decrease in the abundance of this hemicellulose- and cellulose-degradation-related enzymes may be caused by substrate dependence, corresponding to the low hemicellulose and cellulose in the LC diets. However, only one oligosaccharide-degrading enzyme—GH32—was more abundant in the HC group compared with the LC group. Since starch degrades faster than fiber, it seems that starch degradation is more dependent on substrate contents rather than drastically adjusting the microbial functions. This may be one of the reasons for the above phenomenon; on the other hand, the small sample size in the present study may also mask some differences.

### HC Diets Altered Microbial Functions Related to the Metabolism of Methane, Lipid, and Amino Acids in the Hindgut of Dairy Cows

Corresponding to the changes in the abovementioned cellulolytic bacteria and methanogens, we found the KEGG pathway—methane metabolism—was enriched in the LC group when compared with the HC group. Subsequently, the visualization results of the KEGG mapper showed that 67 KOs significantly affected by HC diets were related to methane metabolism in the present study. The main methanogenesis pathways in the gastrointestinal tract of livestock animals include the hydrogenotrophic route (predominant), the acetoclastic route, and the methylotrophic route (24). Our results showed that the relative abundance of 10 main microbial genes (*Fdh, Fmd, Ftr, Mch, Mtd-Hmd, Mer, Mtr, Mcr, Frh*, and *Hdr*) related to the hydrogenotrophic route and 3 microbial genes related to the acetoclastic route (*Ack, Mtr*, and *Mcr*) were decreased by HC diets in the present study; as for the methylotrophic route, 4 microbial genes (*MtaB, MtaC, MttB*, and *Mcr*) were decreased, while 1 microbial gene (*MttC*) was increased by HC diets in the present study. *Mcr*, namely, methyl-coenzyme M reductase, the final and rate-limiting step enzyme for catalyzing the methane biogenesis, was shared with these three important methanogenesis routes (24). The decrease in the relative abundance of these microbial genes was involved in methanogenesis pathways further substantively proved that HC diets abled to reduce the methane production from dairy cows. In addition, among the abovementioned changed methanogens, *Methanobrevibacter* utilized hydrogen, and/or formate for methanogenesis, *Methanosphaera* utilized methyl-containing compounds (such as methanol and methylamine) to produce methane, and *Methanosarcina* harbored the abovementioned three methane methanogenesis pathways (41, 42). Hence, in the present study, HC feeding resulted in a decrease in the methanogens populations and gene expressions related to methanogenesis, which further resulted in a reduction in methane production.

Our results also found that the KEGG pathway—Lipid metabolism (level 2) and its sub-pathways included fatty acid metabolism, fatty acid biosynthesis, biosynthesis of unsaturated fatty acids, linoleic acid metabolism, primary bile acid biosynthesis, and secondary bile acid biosynthesis (level 3)—was all enriched in the HC group. Previous studies showed that the biohydrogenation process of poly-unsaturated fatty acids (PUFA) in the rumen was weakened when the HC diets were fed (43, 44). This response was considered to be related to a decrease in cellulolytic bacterial populations and pH value, further causing a reduction in the hydrogen supply of ruminal biohydrogenation (43, 45). Hence, these changes in the rumen may increase the amounts of unsaturated fatty acids entering the lower intestine. Furthermore, studies on pigs and mice confirmed that increasing the starch contents in the hindgut could regulate the lipid metabolism and increase the linoleic acid contents in the hindgut (46, 47). In addition, the reduction in the cellulolytic bacterial populations in hindgut in the present study may also exacerbate the above changes. For a deeper understanding, we screened the differential microbial genes involved in lipid metabolism. A decrease in the abundance of genes *Acd, EchA*, and *Fadj* and an increase in the abundance of genes *AccA, AccB, AccC*, and *PldA* were observed in the HC group when compared with the LC group in the present study. The microbial genes *Acd, EchA*, and *Fadj* were the main enzymes of the β-oxidation pathway in the mitochondrial matrix, and each cycle of this β-oxidation resulted in the production of acetyl-CoA (48); and the *AccA, AccB*, and *AccC* were the encoding genes of acetyl-CoA carboxylase's subunits, which abled to catalyze the carboxylation of acetyl-CoA to produce malonyl-CoA in the first committed step of the fatty acid synthesis pathway (49). In addition, the gene *PldA* involved in the linoleic acid metabolism and abled to catalyze the lecithin to produce linoleate; and the gene *Cgh*, namely, choloylglycine hydrolase, abled to catalyze the deconjugation of bile acids conjugated with glycine or taurine and further assisted in fatty acids metabolism (50, 51). According to the above analysis, it seems that fatty acid synthesis was enhanced and fatty acid metabolism was weakened, which may cause the accumulation of fatty acids in the hindgut of cows fed HC diets. However, because the fatty acids in the feces were not measured in the present study, this result still needs further confirmation in the future.

In addition, our results also revealed that several KEGG pathways belonging to “Carbohydrate metabolism” and “Amino acid metabolism” were also affected by dietary treatments in the present study. In particular, the pathways “Phenylalanine metabolism,” “Tryptophan metabolism”, and “Glycolysis/Gluconeogenesis” were enriched in the LC group, and the pathways “Phenylalanine, tyrosine, and tryptophan biosynthesis” and “Valine, leucine, and isoleucine biosynthesis” were enriched in the HC group. Previous studies demonstrated that increasing the contents of fermentable carbohydrates reached pig's hindgut, the microbes habited in hindgut tended to preferentially utilize the carbohydrates, and hence weaken the fermentation of amino acids (52, 53). Hence, the increased carbon source in the hindgut of dairy cows fed HC diets in the present study may explain the weakened pathways “Phenylalanine metabolism” and “Tryptophan metabolism”, and the enhanced pathways “Phenylalanine, tyrosine, and tryptophan biosynthesis” and “Valine, leucine, and isoleucine biosynthesis”. In addition, the aromatic amino acids phenylalanine and tryptophan are both glucogenic and ketogenic amino acids (54), and their weakened metabolism and sufficient glucose supply during HC feeding may partly contribute to weakening the pathway “Glycolysis/Gluconeogenesis” in the HC group in the present study.

## Conclusion

**Figure 7:**
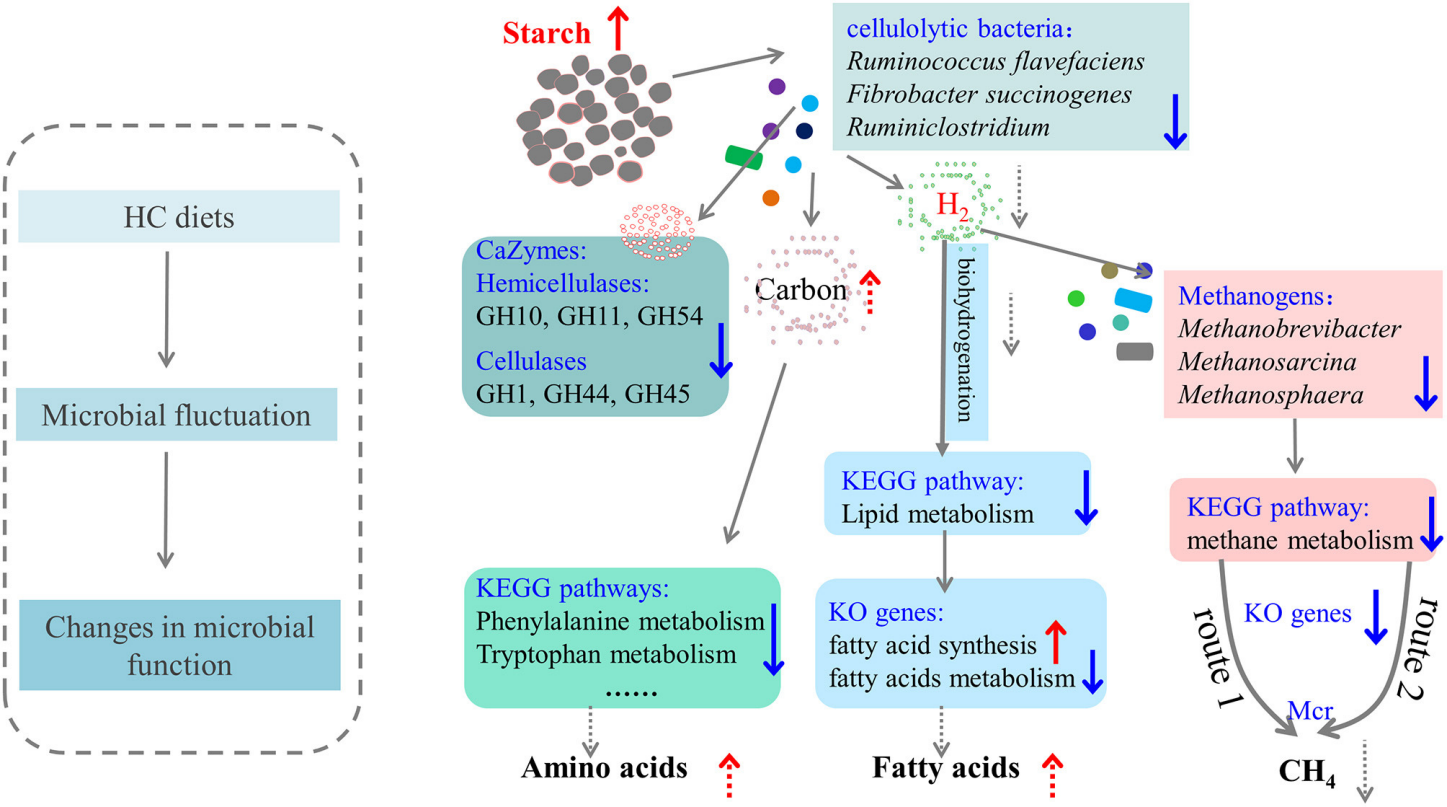
The proposed schematic diagram of how high-concentrate (HC) diets affected the hindgut microbiota and metabolic functions of dairy cows. The up arrows indicated this microbial taxa or function increased or enhanced in the HC group compared with the low-concentrate group. The dotted line was the speculated concept based on these results in the present study, which needed to be confirmed by future studies.

In conclusion, a metagenomics approach was applied to reveal the changes in hindgut microbiota and their metabolic functions of dairy cows. The proposed schematic sketch of how HC diets affected hindgut microbiota and their metabolic functions of dairy cows is mapped in Figure 7. Overall, during HC feeding, hindgut microbiota, especially cellulolytic bacteria (such as *Fibrobacter*, and *Ruminococcus*) and methanogens (such as *Methanobrevibacter, Methanosarcina*, and *Methanosphaera*), was decreased and correspondingly resulted in a decrease in the relative abundance of CAZy related to hemicellulases (GH10, GH11, and GH54) and cellulases (GH1, GH44, and GH45). Furthermore, the KEGG pathways “Methane metabolism” (enriched in the LC group), “Lipid metabolism” (enriched in the HC group), and several sub-pathways in “Amino acid metabolism” (such as Phenylalanine metabolism, Phenylalanine, tyrosine, and tryptophan biosynthesis) of the hindgut microbiota of dairy cows were all affected by HC diets. Therefore, it may be reasonable to speculate that, HC diets caused the remodeling of hindgut microbiota, giving priority to utilize carbon sources, reducing the decomposition of amino acids and methane production, and increasing the accumulation of fatty acids. However, more studies on how HC diets affected hindgut microbiota with larger sample sizes and hindgut metabolites are required.

## Data Availability Statement

The datasets presented in this study can be found in online repositories. The names of the repository/repositories and accession number(s) can be found below: https://www.ncbi.nlm.nih.gov/, PRJNA641261.

## Ethics Statement

The animal study was reviewed and approved by Nanjing Agricultural University Animal Care and Use Ethics Committee.

## Author Contributions

SM and XW: designed the present experiment. RZ and JL: performed the experiment, analyzed the data, and wrote the manuscript. SM, XW, and LJ: revised the manuscript. All the authors had read and approved the final manuscript.

## Funding

This study was funded by the Fundamental Research Funds for the Central Universities (JCQY201905), the Open Project of Beijing Key Laboratory of Dairy Cow Nutrition, Beijing University of Agriculture, China, the Fundamental Research Projects of Colleges and Universities of Liaoning Province of China (No. LSNQN201706), and the National Natural Science Foundation of China (No. 31360558).

## Conflict of Interest

The authors declare that the research was conducted in the absence of any commercial or financial relationships that could be construed as a potential conflict of interest.

## Publisher's Note

All claims expressed in this article are solely those of the authors and do not necessarily represent those of their affiliated organizations, or those of the publisher, the editors and the reviewers. Any product that may be evaluated in this article, or claim that may be made by its manufacturer, is not guaranteed or endorsed by the publisher.
