# Supplementary material for: The Remodeling Effects of High-Concentrate Diets on Microbial Composition and Function in the Hindgut of Dairy Cows
Source: Front Nutr. 2022 Feb 1;8:809406. doi: 10.3389/fnut.2021.809406 (PMC8845480; doi:10.3389/fnut.2021.809406)
Supplement: Supplementary file 1 [file Image_1.pdf]

Supplementary Material

Supplementary Tables

Supplementary Table 1 Ingredients and chemical composition of diets.

Ingredients (% of DM)[a]	LC	HC
Alfalfa hay	27.00	12.00
Leymus chinensis	3.00	4.00
Straw	0.00	4.00
Maize silage	30.00	10.00
Maize	20.00	45.00
Wheat bran	0.00	1.40
Soybean meal	10.85	15.00
cottonseed meal	6.00	5.00
Calcium carbonate	0.40	0.85
Calcium hydrogen phosphate	0.75	0.75
Sodium bicarbonate	0.75	0.75
Sodium chloride	0.50	0.50
Dairy premix[b]	0.75	0.75
Nutrient composition (% of DM)		
NEL(Mcal kg^{-1} of DM)	1.54	1.71
Crude protein (CP)	15.8	15.8
Neutral detergent fiber (NDF)	34.9	24.7
Acid detergent fiber	23.0	13.2
Non-fiber carbohydrates (NFC)	36.1	46.3
Calcium	0.88	0.82
Phosphorous	0.51	0.53

[a] Fed as TMR.

[b] Contained Mn, 0.24%; K, 0.5%; S, 0.2%; Zn, 4,000 mg kg^{-1}; Cu, 1,000 mg kg^{-1}; Mn, 2,500 mg kg^{-1}; I, 64 mg kg^{-1}; Co, 5 mg kg^{-1}; vitamin A, 1,000,000 IU kg^{-1}; vitamin D, 110,000 IU kg^{-1}, and vitamin E, 6000,000 IU kg^{-1}.

LC= low concentrate diet, HC= high concentrate diet

Supplementary Figures

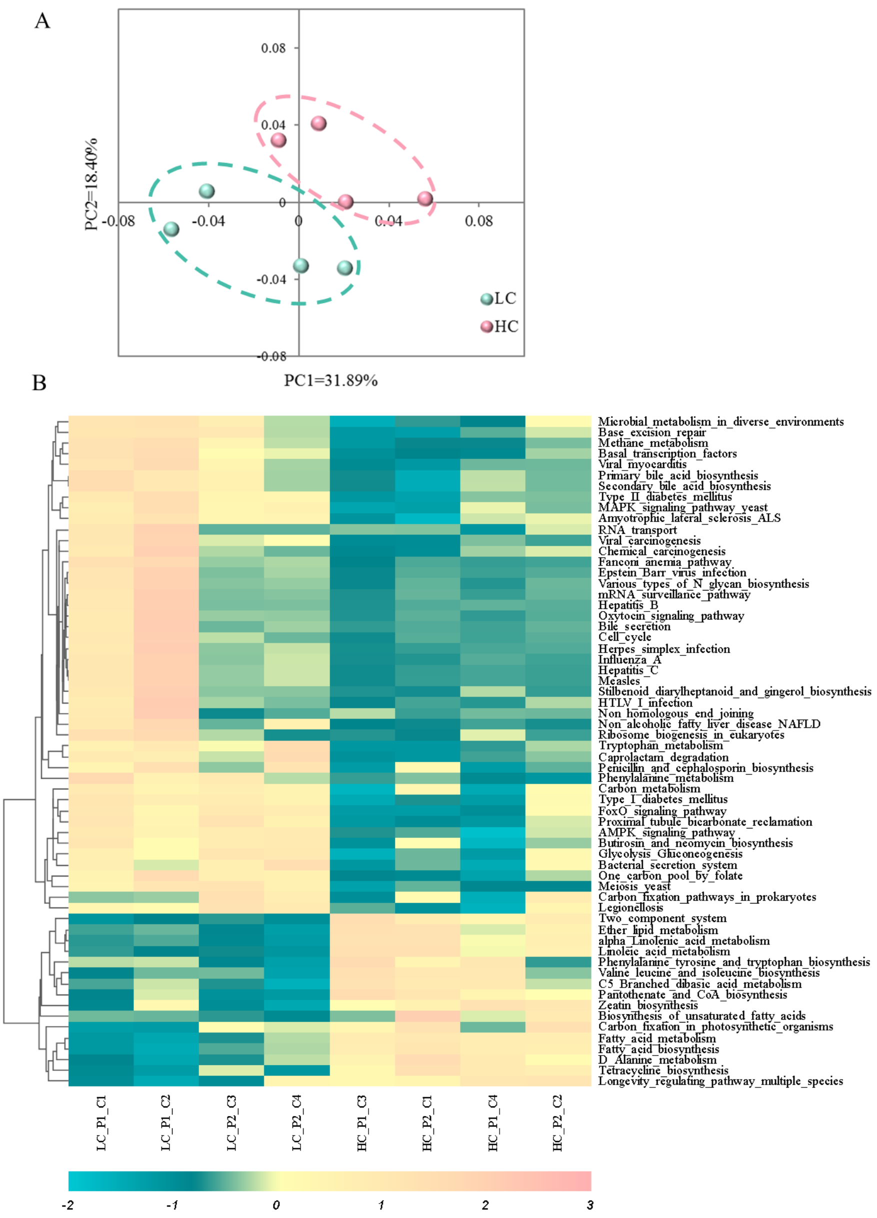

Supplementary Figure 1 Effects of high-concentrate (HC) diets on KEGG profiles of fecal microbiota of dairy cows. (A) The principal coordinate analysis (PCoA) of fecal microbial functions at KEGG pathway level 3; (B) comparison of microbial functions at KEGG level 3 based on Bray-Curtis dissimilarity matrix.

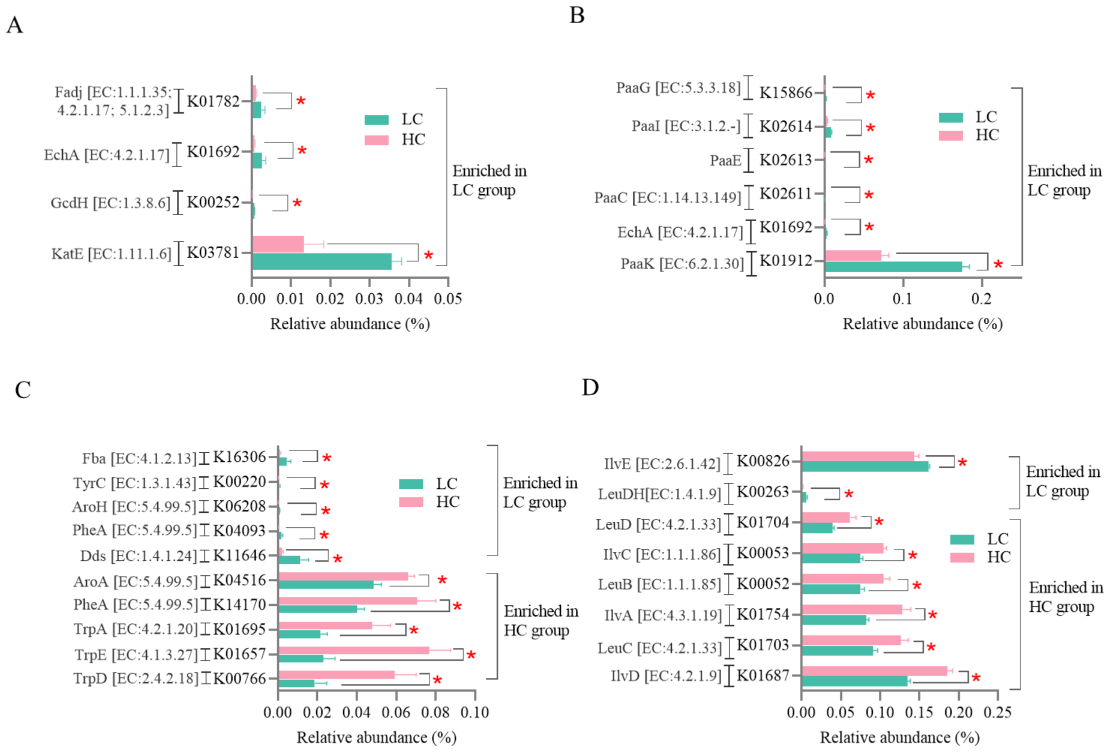

Supplementary Figure 2 Effects of high-concentrate (HC) feeding on microbial genes involved in amino acid metabolism in the hindgut of dairy cows. Only the affected pathways related to amino acids were presented.

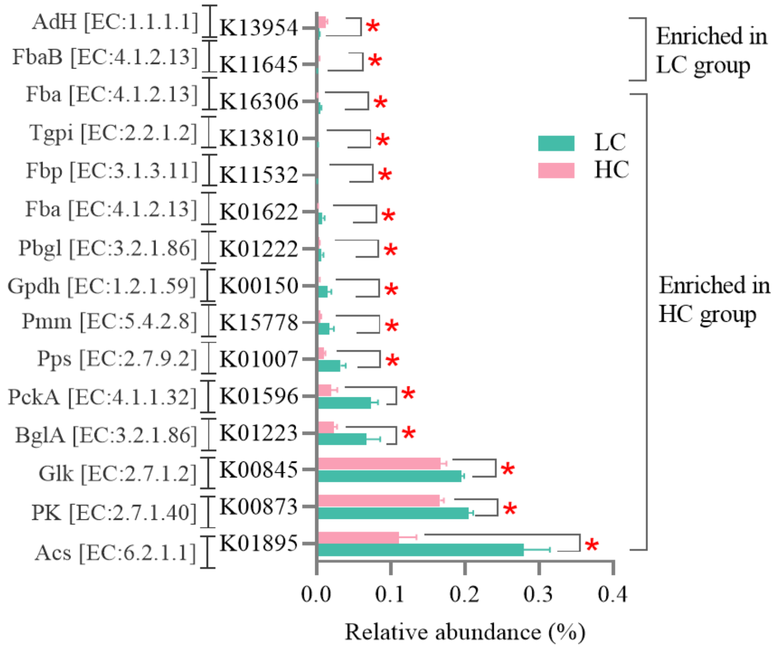

Supplementary Figure 3 Effects of high-concentrate (HC) feeding on microbial genes involved in the pathway—Glycolysis/Gluconeogenesis in the hindgut of dairy cows.
